# Sustained Decrease in Oxygen Saturation in Human Fibular Fractures Monitored with Laser-Doppler and White-Light Spectroscopy: A Longitudinal Observational Pilot Study

**DOI:** 10.3390/jfb17060306

**Published:** 2026-06-22

**Authors:** Tokio Kawamura, Selma Fensel-Merz, Marcel Orth, Emmanouil Liodakis, Yohei Yanagisawa, Bergita Ganse

**Affiliations:** 1 Department of Experimental Musculoskeletal Medicine, Clinics and Institutes of Surgery, Saarland University, 66421 Homburg, Germany; tokio.kwmr@tsukuba-seikei.jp (T.K.); selma.fensel@uks.eu (S.F.-M.); 2 Department of Orthopaedic Surgery, Institute of Medicine, University of Tsukuba, 1-1-1 Tennodai, Tsukuba 305-8575, Ibaraki, Japan; yanagisawa@tsukuba-seikei.jp; 3 Graduate School of Medicine, University of Tsukuba, Tsukuba 305-8575, Ibaraki, Japan; 4 Department of Trauma, Hand and Reconstructive Surgery, Saarland University Hospital, 66421 Homburg, Germany; marcel.orth@uks.eu (M.O.); emmanouil.liodakis@uks.eu (E.L.)

**Keywords:** nonunion, perfusion, bone, oxygenation, blood flow, human physiology, orthopedic surgery, smart implant

## Abstract

Noninvasive light-based measurements have recently been suggested for monitoring fracture healing and for the development of smart implants. The aim of this study was to collect the first exploratory longitudinal *in vivo* data from human distal fibular fractures. In this prospective observational pilot study, blood flow, oxygen saturation, and relative hemoglobin were noninvasively measured by using combined laser Doppler and white-light spectroscopy at depths of 3 mm and 10 mm. In patients with fibular fractures, measurements were performed at 1–3 days, 2 weeks, 6 weeks, 3 months and 6 months after surgery. Patients with fibular nonunion and healthy control participants underwent a single measurement. Fourteen longitudinal fracture patients, a nonunion patient, and 42 controls were included. In the longitudinal fracture group, oxygen saturation at a depth of 10 mm significantly decreased from baseline to 2 weeks (*p* < 0.001) and remained at a low plateau significantly below healthy control levels throughout the 6-month period. Blood flow and relative hemoglobin levels did not longitudinally change but remained significantly elevated compared with controls (*p* < 0.001). A single nonunion case demonstrated a markedly low oxygen saturation value (8.3%) combined with increased blood flow. Fibular fractures treated with plate fixation exhibit a prolonged low-oxygen saturation plateau, in contrast to the rapid recovery observed in tibial shaft fractures, possibly due to differences in anatomy or healing mechanisms. The low oxygen saturation observed in the nonunion requires further investigation, as it may have prognostic potential.

## 1. Introduction

Nonunion of bone fractures is a frequent complication demonstrating a high socioeconomic burden [1]. In clinical research and practice, bone healing progress is still predominantly monitored by using radiographic imaging combined with subjective clinical impressions, despite their well-known limitations. The development of better diagnostic and prognostic alternatives to identify patients who are at risk of developing nonunion at early points in time is highly important. Among the recently identified possible alternatives are spectroscopy-based methods that allow for the non-invasive monitoring of changes in blood flow (BF) and oxygen saturation (SO_2_) in the fracture gap during the healing process [2,3]. These options are of particular interest for the development of smart implants, either as an on-board sensor, or to quantify the benefits of smart implants over conventional ones in clinical studies [2,3]. Smart implants are currently being developed to provide mechanical or chemical sensors directly at the fracture site. In some cases, even actuation via the smart implant may be beneficial, such as by changing the stiffness of the implant depending on the individual biomechanical demand. Smart implants are particularly desirable, as they allow continuous live monitoring of the healing progress.

Radiographic assessment of bone union is associated with a diagnostic delay, as it provides reliable detection of bone healing only after mineralization [1]. Furthermore, a definitive nonunion diagnosis is not always attainable, particularly when orthopedic implants obscure the view of the fracture site. These limitations frequently necessitate supplementary computed tomography (CT) imaging, which elicits concerns regarding increased radiation exposure [4,5]. A nonunion is typically diagnosed when a fracture demonstrates no signs of healing after a period of 6 months or more, accompanied by symptoms such as pain, which often require further nonsurgical or surgical interventions [6]. Nonsurgical treatment options for nonunion include methods such as prolonged immobilization, nutritional optimization, smoking cessation [7], and the application of external stimulation, such as low-intensity pulsed ultrasound (LIPUS), extracorporeal shock-wave therapy (ESWT) or magnetic field therapy [8,9,10]. Surgical interventions for nonunion are broadly classified into two categories, including those that provide additional stability (e.g., exchange nailing) and those that provide biological augmentation (e.g., autologous bone grafting). However, the application of these treatments requires a confirmed diagnosis, which is usually the cause of the significant clinical time lag. To address this challenge, a monitoring method that can predict bony union earlier than conventional approaches is needed [11]. The early identification of nonunion risk would facilitate earlier therapeutic intervention, thus reducing the duration of disability and healthcare costs [12]. Furthermore, for such a method to be clinically viable, it should be simple and noninvasive.

In tibial fractures, recent research has demonstrated that bone healing can be monitored *in vivo* via combined laser Doppler and white-light spectroscopy [3]. This technology can demonstrate characteristic temporal trajectories in SO_2_ and BF at the fracture site throughout healing [3]. Surprisingly, in contrast to previous animal studies [13,14], this technology demonstrated that SO_2_ did not immediately decrease to its lowest level. Instead, it slowly decreased to a minimum level at 2 to 3 weeks after the operation, followed by a subsequent increase. Concurrently, the study demonstrated that BF increased to a maximum level that likewise occurred after 2 to 3 weeks before it decreased. In addition, the SO_2_ values in the nonunion group were similar to the minimum values observed in the longitudinal fracture group, thus indicating insufficient oxygenation in at least some of the cases. Thus, the SO_2_ concentration and BF detected in the fracture seem to be suitable parameters for monitoring fracture healing and possibly even for predicting nonunion. Notably, the authors obtained a similar trajectory of oxygenated hemoglobin in the fracture gap, with a minimum being observed after 2 to 3 weeks when using near-infrared spectroscopy (NIRS) in another longitudinal clinical observational trial studying tibial fractures [2]. Another previous study revealed an inverse relationship of tibial fracture SO_2_ levels with the dosage of norepinephrine administered in unstable intensive care unit patients [15], which demonstrated α-receptor-transmitted vasoconstriction of the bone nutrient vessels, thereby proving that measurements truly capture the bone status and not solely soft tissue oxygenation.

Ankle fractures are among the most common fractures observed in humans [16]. Distal fibular fractures are typically classified by using the Weber classification system [17]. Although many Weber A fractures and a subset of Weber B fractures are amenable to conservative management, many Weber B fractures and most Weber C fractures typically necessitate surgical intervention through open reduction and internal fixation (ORIF). ORIF with plate fixation remains the predominant surgical approach for distal fibular fractures [18], thus providing absolute stability and aiming for primary bone healing. In contrast, intramedullary nailing for treating tibial shaft fractures provides relative stability and leads to secondary bone healing. In contrast, intramedullary nailing for tibial shaft fractures provides relative stability and leads to secondary bone healing.

Anatomically, the fibula is considerably thinner than the tibia, with a reported medullary diameter of 3.2 mm (±1.2) being observed at the malleolus [19]. It also contains a comparatively smaller proportion of cancellous bone. Although the fibula contributes to stabilizing the ankle joint, it bears only a minimal load compared with the tibia, which serves as the primary weight-bearing structure. However, in cases of a combined fracture of the lateral malleolus and tibial shaft, the stability of the fibula significantly influences the mechanical environment of the tibia, particularly regarding the stresses imposed on the tibial fracture site and the intramedullary nail [20].

Furthermore, the fibular malleolus benefits from a rich blood supply, which is primarily derived from branches of the peroneal artery. Significant contributions also originate from the perimalleolar arterial ring, which is formed by anastomoses between the anterior tibial, posterior tibial, and peroneal arteries [21,22]. Collectively, these distinct anatomical, mechanical, and vascular characteristics are thought to contribute to the generally faster healing rates and lower incidence of nonunion observed in fibular fractures than in tibial shaft fractures.

Based on the abovementioned findings, the primary hypothesis was that the longitudinal trajectory of fibular fracture oxygenation reaches its minimum level at 2 to 3 weeks after surgery, followed by a subsequent increase. The secondary hypotheses were 1. a BF increase to a maximum level that is likewise reached after 2 to 3 weeks, followed by a decline, and 2. no changes in rHb.

## 2. Materials and Methods

Ethical approval for this exploratory human *in vivo* longitudinal observational cohort study was obtained from the institutional review board of the Saarland Medical Board (Ärztekammer des Saarlandes, Saarbrücken, Germany; application number 127/22). Written informed consent was obtained according to the Declaration of Helsinki prior to commencement of the study. This study was registered with the German Clinical Trials Register (DRKS00035253). Participation in this purely observational study did not affect the patients’ treatments.

### 2.1. Patient Recruitment and Follow-Up

Patients aged 18 years or older who sustained a distal fibular fracture (Weber B type/AO Classification 44-B type) and underwent ORIF at Saarland University Hospital between October 2024 and July 2025 were prospectively recruited for this study. The inclusion criteria were the ability to provide written and verbal informed consent; ages of 18 years and older; the presence of a distal fibular fracture (AO 44-B) treated with ORIF; and the willingness of the patient to participate in follow-up measurements for at least 6 weeks after surgery. The exclusion criteria were the inability to provide written and verbal informed consent; ages younger than 18 years; and the presence of an open fracture or the patient being polytraumatized.

The first measurement was conducted within 1 to 3 days after surgery (T0). Subsequent measurements were performed at approximately 2 weeks (T1), 6 weeks (T2), 3 months (T3), and 6 months (T4) after surgery, aligning with scheduled follow-up appointments. For internal comparison, perfusion of the uninjured contralateral fibula was measured once at either 3 or 6 months after surgery, in order to minimize the potential influence of trauma-related systemic inflammation. Standard radiographs were obtained at 6 weeks and 3 months after surgery in all the cases. If a syndesmotic screw was used to treat syndesmotic rupture, it was routinely removed after 6 weeks. In addition, patients with a distal fibular fracture without the occurrence of fracture union at 6 months after surgery were recruited for a single measurement from the nonunion outpatient clinic by using the same inclusion and exclusion criteria.

After the surgery, patients usually received enoxaparin as an anticoagulant, and ibuprofen, paracetamol/acetaminophen and in some cases metamizole as pain medications, combined with pantoprazole for stomach protection. Medications were adapted to the individual risk factors and needs. Some of the patients already had chronic medications due to pre-existing conditions.

### 2.2. Healthy Control Group

To provide a baseline for comparison, healthy control participants were recruited. A single measurement was performed at the distal fibula for each control subject. The reason why only a single measurement was performed rather than longitudinal measurements is that without a fracture, no longitudinal changes were expected. The inclusion criteria were the ability to provide written and verbal informed consent; ages of 18 years and older; and no history of fracture or surgery at the measurement site. The exclusion criteria were the inability to provide written and verbal informed consent; ages younger than 18 years; and a history of previous fracture or surgery at the measurement site.

### 2.3. Measurements

The SO_2_, relative hemoglobin amount (rHb), and BF at the fracture site were measured by using a CE-certified medical device that utilizes laser Doppler and white-light spectroscopy (‘oxygen to see’, O2C, LEA Medizintechnik, Gieβen, Germany). The O2C uses a laser with a wavelength of 820 nm and an emitter/detector in the 500–800 nm range of the white-light spectrum. The device calculates BF via the laser Doppler shift and determines SO_2_ and rHb by analyzing the hemoglobin absorption spectra. Black kinesiology tape (BB Sport GmbH & Co. KG, Töging am Inn, Germany) was used to noninvasively adhere probes to the skin to adjust for ambient light and standardize contact pressure. By applying pressure to the kinesiology tape adjacent to the probe rather than the probe itself, the probes were uniformly secured. Single-use polyurethane “Ultracover for TEE Endocavity Probe Cover” (ECOLAB, Microtek Medical B.V., Zutphen, The Netherlands) was used to cover the probes. All of the measurements were performed by using probes with fixed measurement depths of 3 mm and 10 mm. A depth of 3 mm was selected to assess the skin and soft tissue perfusion at the fracture site, whereas a depth of 10 mm targeted the bone tissue. Unless otherwise specified, data obtained from the 10 mm depth are discussed as the primary outcome. The reason why measurements were not only conducted in the bone (10 mm depth) but also in the skin and soft tissue (3 mm depth) is to demonstrate that the observations are bone-specific and not related to changes in the skin and soft tissue vasculature. In addition, general perfusion deficits, such as due to peripheral arterial disease, would similarly show up in the 3 mm measurement, which improves the ability to interpret the findings correctly.

All of the measurements were performed with the participant in the supine position. The fracture position was confirmed by measuring the distance from the tip of the fibula using a tape measure based on the exact fracture location in the radiographs. To avoid interference from the osteosynthesis plate (which was typically placed either posterolaterally or laterally), all of the measurements were consistently performed at the anterolateral aspect of the fibula. At each time point, three individual measurements were acquired from slightly different positions around the fracture site, that differed by a few millimeters only along the fracture gap. Each individual measurement was recorded for 10 s, after which the raw data were averaged by using the device’s internal software. The averages of the three values were subsequently again averaged and the final average was then used for the statistical analyses. For the uninjured contralateral fibula, data were collected at 3 or 6 months after surgery at the corresponding level of the injured side by utilizing the same probes. Moreover, for the healthy control group, measurements were conducted at a standardized location (5 cm from the tip of the distal fibula) using identical probes. All of the measurements were performed by a single investigator (T.K.) to ensure consistency.

### 2.4. Statistics

Due to a lack of previous data, a sample size calculation could not be conducted. The patient number was determined based on patient availability between October 2024 and October 2025. This study was conducted as a pilot study. All of the statistical analyses were performed by using JASP (Version 0.95.4; JASP Team, University of Amsterdam, Amsterdam, The Netherlands). The normality of all of the continuous data was assessed by using the Shapiro–Wilk test. Continuous variables are presented as the means ± standard deviations (SDs). A *p* value < 0.05 was considered to indicate statistical significance for all of the tests. As normality was not confirmed in many groups (or due to the small sample size), nonparametric tests were primarily used for group comparisons. A linear mixed model (LMM) was employed to analyze longitudinal changes in SO_2_, BF, and rHb. LMMs are advantageous for longitudinal data as they efficiently handle missing data points, such as loss to follow-up, without requiring data imputation or the exclusion of patients. “Time” was set as a fixed effect and “patient” as a random effect to account for the intra-individual correlation of repeated measures. The *p*-values for the post hoc comparisons between time points following the LMM analysis were adjusted using the Bonferroni correction. This approach allows for the inclusion of all of the available data, thereby resolving missing data points (such as data missing due to loss to follow-up at later time points). Time was used as a fixed effect, and patient was used as a random effect. Post hoc comparisons between time points were conducted by using the “specify contrast” function, and *p* values were adjusted by using the Bonferroni correction. Comparisons between two independent groups were performed by using the independent *t*-Test or the Mann–Whitney U test, and paired groups were analyzed by using the Wilcoxon signed-rank test.

## 3. Results

A total of 14 patients in the longitudinal group, one nonunion patient, and 42 healthy control participants were included (Table 1 and Table 2). All of the longitudinal patients completed the follow-up assessments at T0, T1, and T2 (*n* = 14). Data at T3 and T4 were available for 13 patients and 8 patients, respectively. Compared with the healthy control group, the longitudinal patient group was significantly older and demonstrated a shorter body height. There were no significant differences observed in sex distribution, BMI, smoking habits or the prevalence of diabetes mellitus between the two groups. To assess whether these demographic differences affected the measurement outcomes, Spearman’s rank correlation analysis was performed within the healthy control group. The analysis revealed no significant correlations between either age or body height and any of the measured perfusion parameters (SO_2_, rHb, and BF at both depths) (all *p* > 0.05). Therefore, the demographic differences were considered to be negligible. With respect to clinical outcomes, no cases of implant failure, loss of reduction, or nonunion were observed in any patient during the 6-month observation period. Two cases required external fixation prior to ORIF, but no open fractures were included in the study. A 1/3 tubular plate was used in 10 cases, and an anatomical locking plate was used in 4 cases. Nine cases involved screw fixation of the syndesmosis. There was no standardized rehabilitation protocol. The patients with an isolated fibular fracture were allowed to weight-bear as tolerated using a VACOped boot (OPED GmbH, Valley/Oberlaindern, Germany). For cases involving syndesmosis fixation, only partial weight-bearing was permitted until the syndesmosis screw was removed, though specific details varied by surgeon’s preference.

### 3.1. Differences Between 3 mm and 10 mm in the Control Group

Table 3 shows the average values and SD in the healthy control group comparing 3 mm and 10 mm. The SO_2_ values were significantly lower in 3 mm than in 10 mm. The rHb was significantly greater in 3 mm than 10 mm, while there was no difference in BF between the depths.

### 3.2. Longitudinal Findings

**Figure 1:**
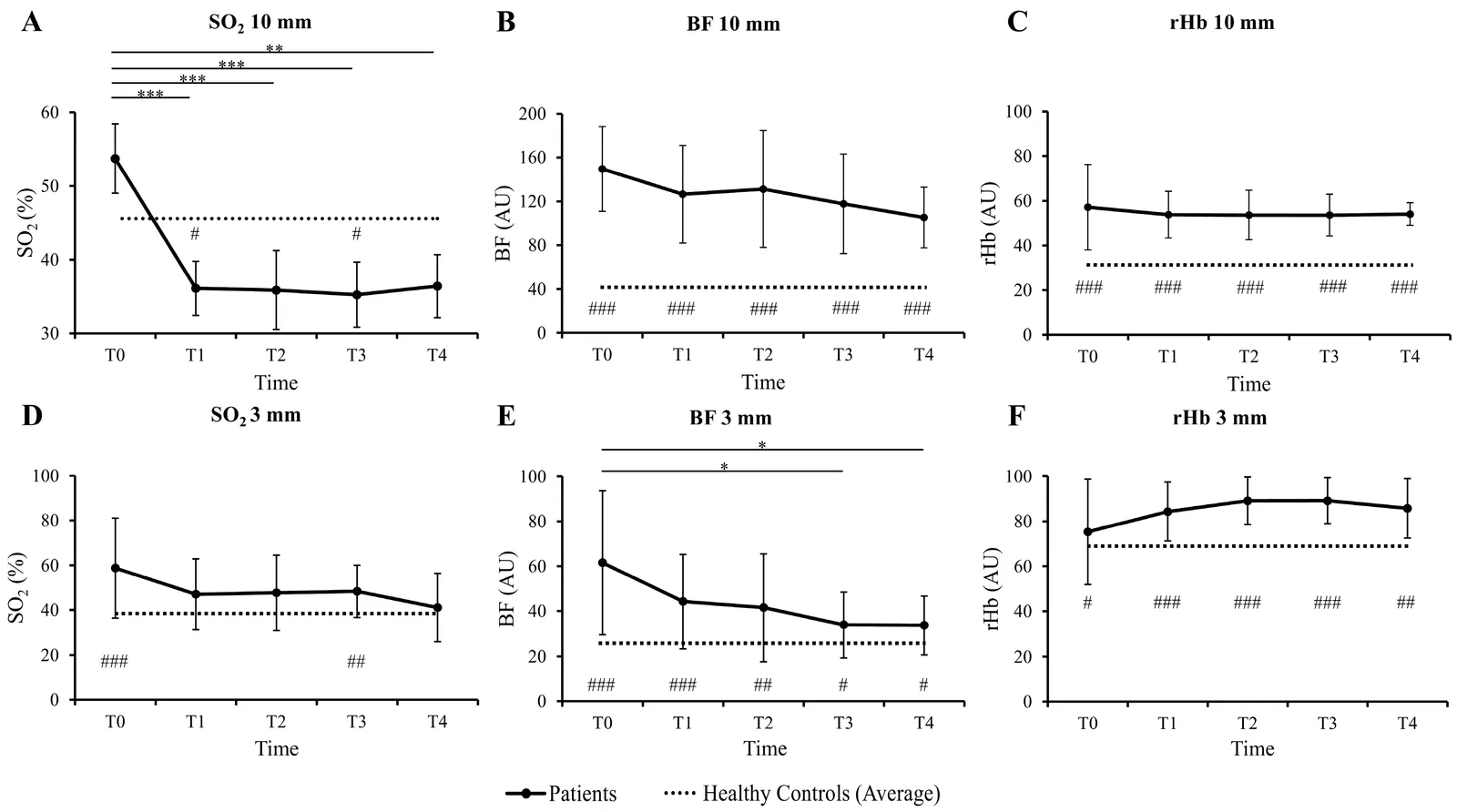
Longitudinal data obtained at depths of 10 mm and 3 mm. Panels (**A**–**C**) show longitudinal changes in oxygen saturation (SO_2_), blood flow (BF), and relative hemoglobin (rHb) at a depth of 10 mm. Panels (**D**–**F**) show the corresponding changes at a depth of 3 mm. The data are presented as the means ± standard errors (SEs) of the observed raw values (*n* = 14 at T0–T2, *n* = 13 at T3, and *n* = 8 at T4). The dotted gray line represents the mean value of the healthy control group (*n* = 42). Statistical significance: * *p* < 0.05, ** *p* < 0.01, *** *p* < 0.001 vs. T0 (linear mixed model); # *p* < 0.05, ## *p* < 0.01, ### *p* < 0.001 vs. healthy controls (Mann–Whitney U test). Time points: T0, 1–3 days; T1, 2 weeks; T2, 6 weeks; T3, 3 months; and T4, 6 months after surgery.

The LMM analysis revealed a significant main effect of time on the 10 mm SO_2_ (F = 6.019, *p* < 0.001). Post hoc comparisons revealed that the SO_2_ values at T0 were significantly greater than those at all of the subsequent time points (T1–T3: *p* < 0.001; T4: *p* = 0.008). Following the initial decrease from T0 to T1, no significant differences were observed between the subsequent time points, thus demonstrating that SO_2_ significantly decreased at T1 and persisted at this lower level throughout the 6-month follow-up. Compared with the healthy control group, SO_2_ was significantly lower at T1 (*p* = 0.034) and T3 (*p* = 0.032) (Figure 1A). In contrast, no significant longitudinal changes were observed for 10 mm rHb (*p* = 0.934) or BF (*p* = 0.145). Compared with those in the healthy control group, BF and rHb were both significantly elevated at all of the time points (all *p* < 0.001) (Figure 1B,C). Figure S1 shows the individual longitudinal SO_2_ trajectories at 10 mm depth.

For the superficial measurements (3 mm depth), a significant main effect of time was observed for BF (F = 3.315, *p* = 0.018), with post hoc tests showing significant decreases from T0 to T3 (*p* = 0.011) and from T0 to T4 (*p* = 0.046) (Figure 1E), whereas no significant longitudinal changes were observed for SO_2_ or rHb (Figure 1D,F). Compared with that in the healthy control group, the BF at 3 mm was significantly elevated at all of the time points (T0–T1, *p* < 0.001; T2, *p* = 0.007; T3, *p* = 0.029; T4, *p* = 0.013; Figure 1E). Similarly, rHb was significantly elevated at all of the time points (T0, *p* = 0.034; T1–T3, *p* < 0.001; T4, *p* = 0.002; Figure 1F). Moreover, SO_2_ was significantly elevated at T1 (*p* < 0.001) and T3 (*p* = 0.008) (Figure 1D).

### 3.3. Cross-Sectional Comparisons

**Figure 2:**
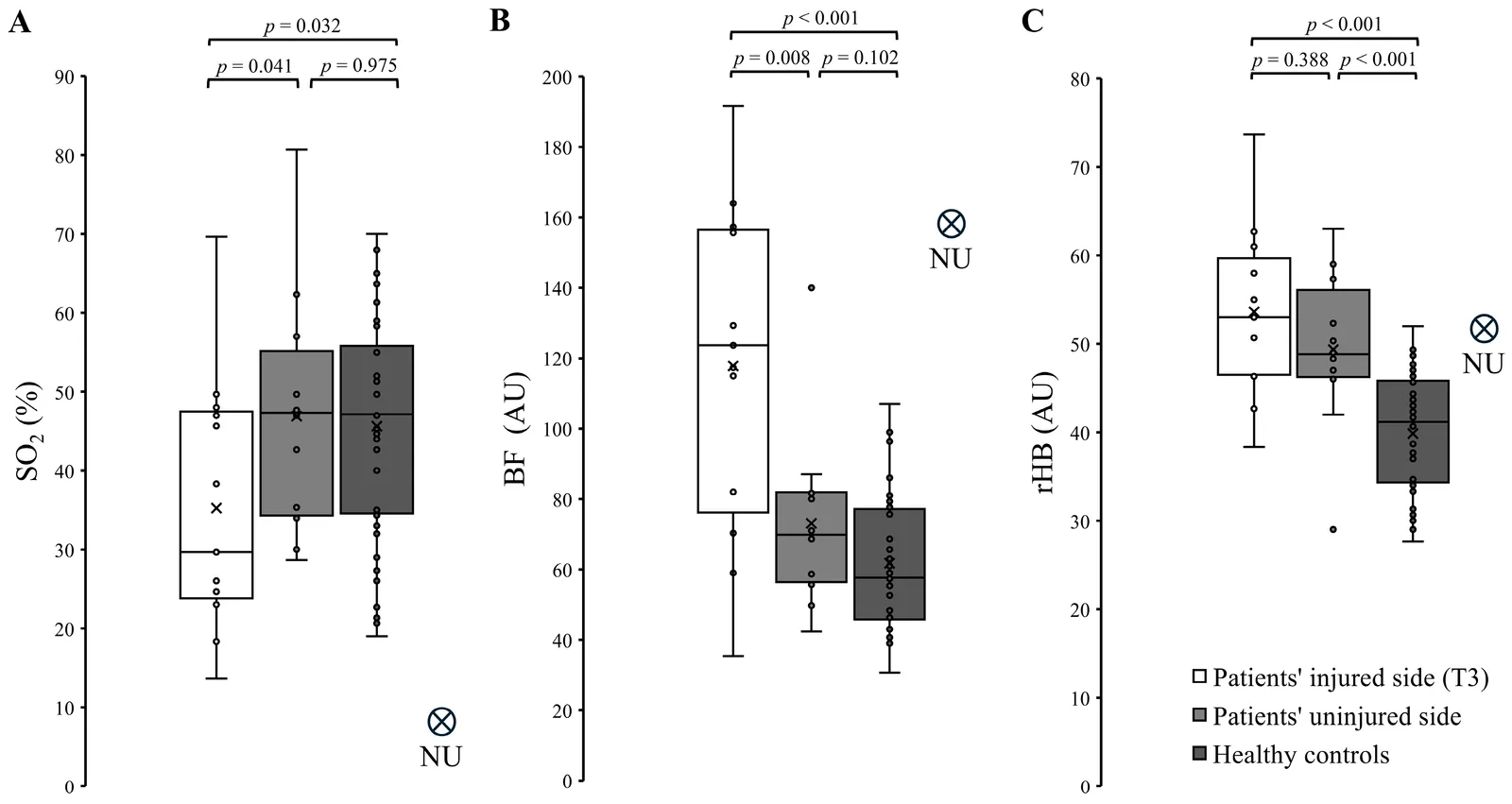
Cross-sectional data at a depth of 10 mm. Box plots showing comparisons between the injured side (3 months after surgery; *n* = 13), uninjured side (3 or 6 months after surgery; *n* = 12), and healthy control group (*n* = 42) for (**A**) oxygen saturation (SO_2_), (**B**) blood flow (BF), and (**C**) relative hemoglobin content (rHb). The data points for the nonunion case are indicated by NU. Boxes represent the interquartile ranges, horizontal lines indicate medians, and whiskers indicate the 1.5 × interquartile ranges (IQRs). Individual data points are plotted. Statistical comparisons were performed by using the Wilcoxon signed-rank test (vs. the uninjured side) and the Mann–Whitney U test (vs. the healthy control group). Exact *p* values are displayed in the figure.

The distributions of SO_2_, BF, and rHB at a depth of 10 mm, comparing the injured side (T3), the uninjured side (individual baseline at T3/T4), and the healthy control group, are shown in Figure 2. Data for the uninjured side were obtained from 12 patients, as 2 patients were lost to follow-up before the measurement (at 3 or 6 months).

With respect to SO_2_, the Mann–Whitney U test revealed that the levels on the injured side were significantly lower than those in the healthy control group (*p* = 0.032). Furthermore, the Wilcoxon signed-rank test revealed significantly lower SO_2_ levels on the injured side than on the contralateral uninjured side (*p* = 0.041). No significant difference was observed between the uninjured side and the healthy control group (*p* = 0.975) (Figure 2A).

In contrast, for BF and rHb, the injured side exhibited significantly higher values compared to the healthy control group (*p* < 0.001; Figure 2B,C). For BF, the uninjured side demonstrated no significant difference compared to the healthy control group (*p* = 0.102), whereas values on the injured side were significantly higher than on the uninjured side (*p* < 0.001) (Figure 2B). However, for rHb, the values of the uninjured side were significantly higher than in the healthy control group (*p* < 0.001), and no significant difference was observed between the injured and uninjured sides (*p* = 0.388) (Figure 2C).

### 3.4. Nonunion Case

**Figure 3:**
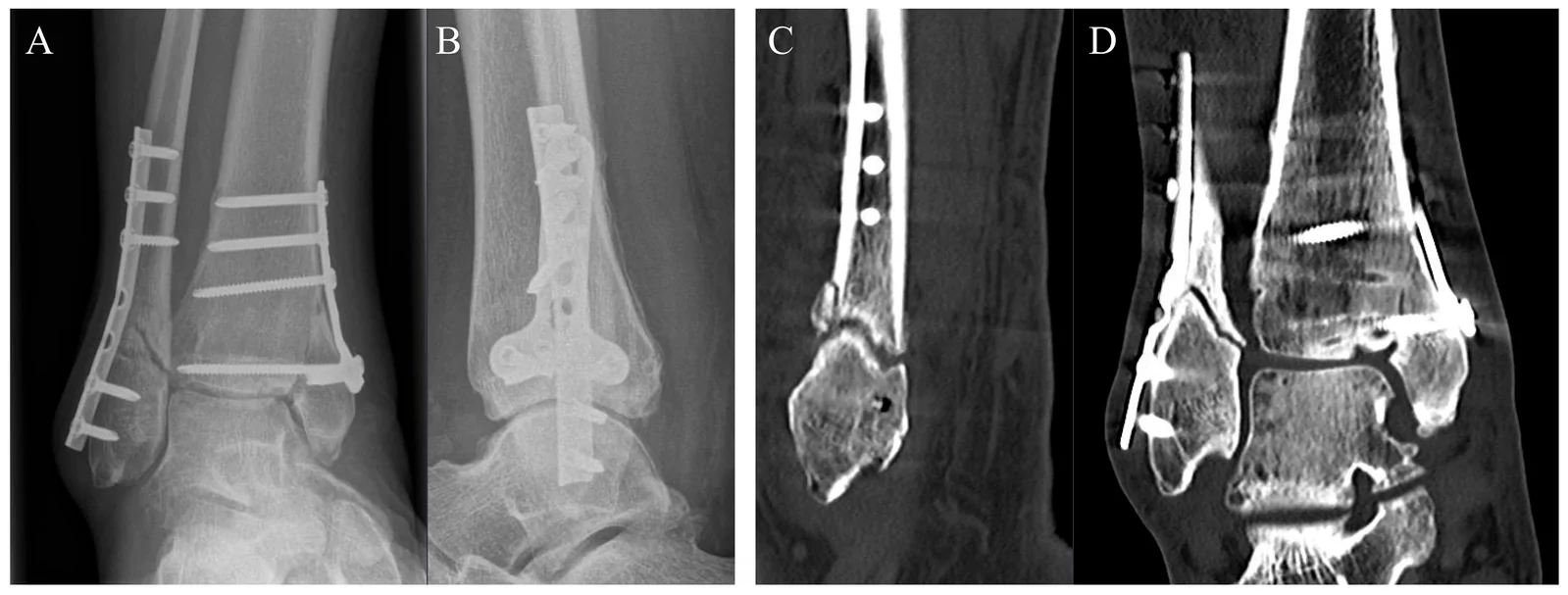
Imaging of the nonunion patient performed 6 months after surgery. Radiographs in (**A**) AP and (**B**) lateral views. CT scans shown in (**C**) sagittal and (**D**) coronal views.

The details of the nonunion patient are provided in Table 2, and the radiographic findings are displayed in Figure 3. The patient underwent surgery (ORIF) at another institution and was subsequently referred to Saarland University Hospital for revision surgery. Measurements were performed 6 months after surgery. The findings revealed a markedly low SO_2_ value of 8.33% at a depth of 10 mm in the fracture, combined with a high BF value of 157.33 AU. The rHb value at 51 AU was not different compared with the longitudinal fracture group (Figure 2). At a depth of 3 mm, BF was elevated (71.67 AU) compared with that in the fracture group, whereas the SO_2_ (47.67%) and rHb (86 AU) did not exhibit significant differences.

## 4. Discussion

This study is the first exploratory pilot study to characterize the longitudinal perfusion trajectories of human distal fibular fractures by using laser Doppler and white-light spectroscopy. In contrast with the primary hypothesis, the SO_2_ exhibited a significant decrease at 2 weeks postoperatively, followed by a sustained plateau that was significantly below the levels of the uninjured side and healthy controls. This plateau persisted throughout the entire 6-month observation period. Second, although neither rHb nor BF demonstrated significant longitudinal fluctuations at a depth of 10 mm, both parameters remained significantly greater than those in the healthy control group. Measurements in the nonunion patient revealed a markedly low SO_2_ value combined with an elevated BF value at a depth of 10 mm.

### 4.1. Trajectory of SO_2_

The initial decrease in SO_2_ in a bone fracture appears to be a universal response to surgical trauma [23], as has been demonstrated in mice [13], sheep [14], and humans [2,3] alike. The finding that the SO_2_ at T0 was not significantly different from that in the control group suggests that the injury or surgery itself was not the immediate cause of the decrease in oxygen saturation. Instead, the subsequent decrease at T1 was likely driven by postoperative edema, coagulation and inflammation, which increase intracompartmental pressure and decrease microvascular perfusion [24]. This initial slow decline is consistent with previous findings observed in human tibial fractures [3], despite differences in anatomy, blood supply and bone structure between the two bones. The comparison of the uninjured side with the injured side revealed lower SO_2_ levels on the uninjured side but no difference from those in the healthy control group. This finding highlights the notion that the decrease in SO_2_ in fractures is likely caused by local processes and not by systemic factors, such as trauma-related inflammation. This theory is supported by the fact that the measured skin and subcutaneous tissue SO_2_ at 3 mm depth did not decrease either. In smaller mammals, hypoxia has been observed to occur much faster compared with the present findings and with data obtained from human tibial fractures [2,3,14]. This discrepancy may be due to differences in the anatomy of the cortical blood supply and blood vessel distribution [25].

**Figure 4:**
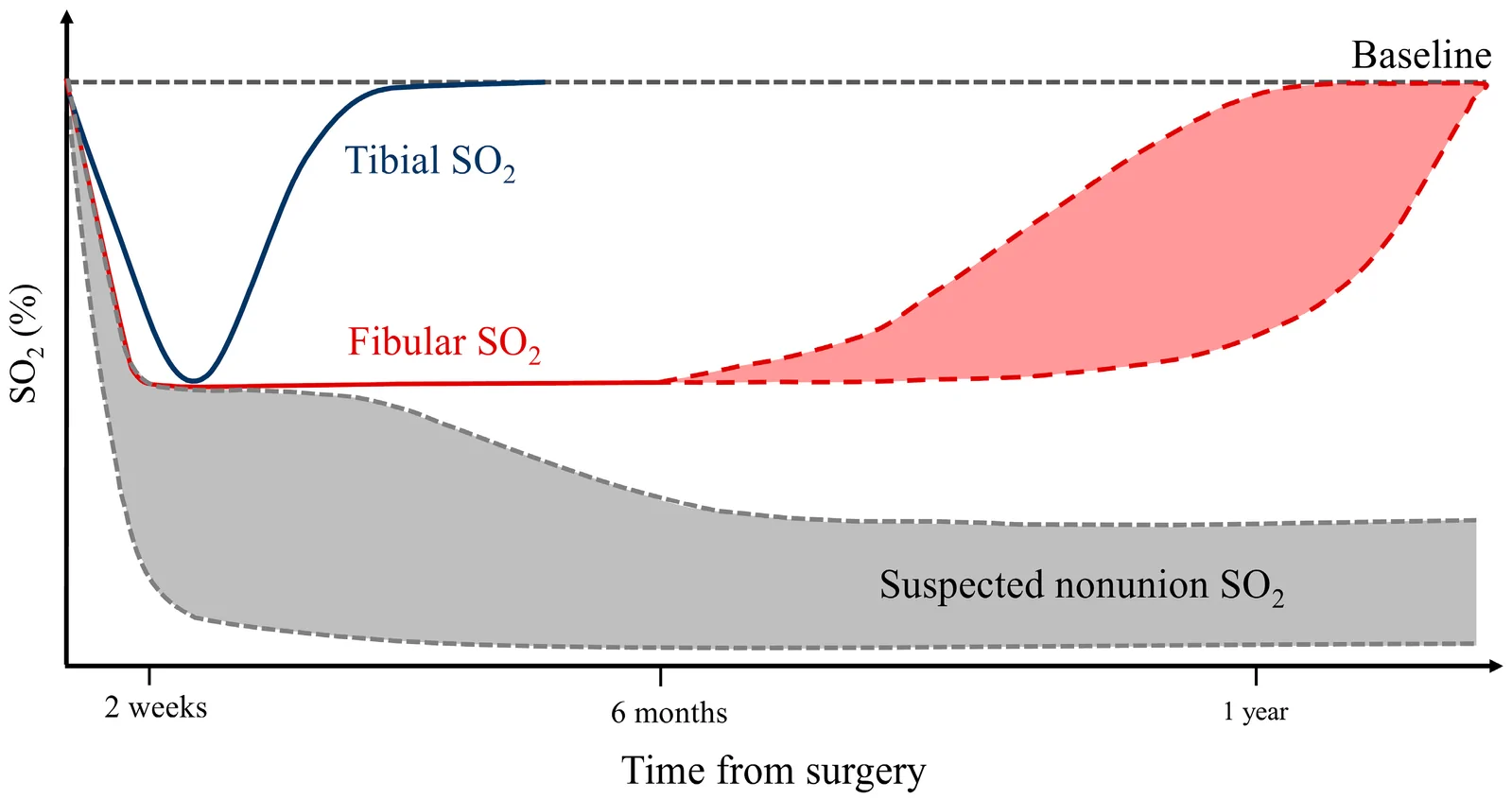
Schematic illustration of SO_2_ trajectories after fracture surgery. The blue solid line represents the decrease and subsequent increase in SO_2_ of the tibial shaft, as demonstrated by Scholz et al. [3]. The red solid line represents the trajectory of the distal fibula SO_2_ (present study), which significantly decreased at 2 weeks after surgery, followed by a sustained plateau. The red shaded region bounded by the dashed lines indicates the hypothesized recovery window beyond 6 months. The gray shaded region bounded by the dashed lines indicates the hypothesized SO_2_ trajectories of hypotrophic nonunion. The horizontal dashed line at the top indicates the baseline or healthy control level.

The subsequent SO_2_ progression observed in the present study markedly contrasts with the decrease and subsequent increase reported for human tibial fractures by Scholz et al. [3], wherein SO_2_ increased after reaching a minimum level (Figure 4). One of the possible explanations for this difference between tibial and fibular fractures involves prolonged changes in fibular bone mineral density and trabecular structures at the former fracture site [26,27]. Although the tibial shaft is characterized by thick cortical bone surrounding the medullary cavity, the distal fibula mainly comprises cancellous bone. Indeed, standard bone oxygen levels are much lower than those in different organs and tissues [28]. In metaphyseal bone, the oxygen tension is lower than that in diaphyseal bone [29]. Further possible differences in the healing mechanisms between the present study and the findings from studies on tibial fractures include differences in the healing mechanisms between relative stability (intramedullary nailing) and absolute stability (ORIF via plating) [30]. During secondary healing under relative stability, extensive callus formation occurs, thus requiring a burst of angiogenesis and high metabolic demand, which drives the decline and subsequent increase in SO_2_ during recovery [31,32]. During secondary fracture healing, cytokines and growth factors lead to vascularization of the fracture within 2–5 weeks [33]. Conversely, under absolute stability achieved via plating, callus formation is suppressed, and bone healing proceeds via primary cortical healing (Haversian remodeling) [30,34]. This process is relatively quiescent compared with secondary healing and does not require a marked angiogenic burst; thus, SO_2_ may not increase again as quickly as it does in secondary healing but possibly persists at a lower plateau [35].

An additional potential explanation for the sustained decrease in SO_2_ levels observed in this study is the impact of the surgical procedure itself. Although intramedullary nailing preserves the periosteal blood supply [36], ORIF via plate fixation necessitates periosteal stripping, thus physically severing the primary oxygen supply route to the cortical bone [37,38]. Although BF was elevated (thus indicating active hyperemia), the structural compromise of the periosteal network likely created a supply–demand mismatch, thereby resulting in sustained local hypoxia (low SO_2_) despite increased blood flow. This “low plateau” state suggests that the fracture site (even after primary bone healing is achieved) does not immediately return to its preinjury physiological baseline, at least within the 6-month follow-up period. It is conceivable that SO_2_ levels may slowly normalize to contralateral values over a longer time period (months to years) during the final remodeling phase (Figure 4).

### 4.2. Blood Flow and Relative Hemoglobin Content

In the present study, neither BF nor rHb demonstrated significant longitudinal fluctuations at a depth of 10 mm. However, compared with those of healthy controls, both parameters remained significantly elevated, thus indicating hyperemia of the fracture site. The 3 mm BF slowly decreased over the course of the 6-month investigation period. The finding that no longitudinal changes in BF were observed at a depth of 10 mm differs from the longitudinal data reported in human tibial fractures, wherein BF was observed to increase to a maximum level, followed by a decline and a return to normal values [3]. An increase in BF followed by a decrease has similarly been described in dogs with tibial osteotomies, wherein BF returned to a normal level after 12 weeks [39]. Similar BF trajectories have been reported for tibial osteotomies in rabbits [40]. The lack of longitudinal changes detected in the rHb finding at a depth of 10 mm may be explained by the anatomical and healing differences in cancellous versus cortical bone, with a pronounced trabecular structure being observed in the distal fibula compared with the thick cortical bone of the tibial shaft. Likewise, aspects of the surgical procedure or the type of fixation may have affected these results.

### 4.3. Differences Between 3 mm and 10 mm

In this study, measurements were not only conducted in the bone (10 mm depth), but also in the skin and soft tissue (3 mm depth) above the bone in the same location. The average of the healthy control group was higher at 10 mm than at 3 mm for SO_2_ and BF, and lower for rHb (Table 3, Figure 1). After fracture, at 10 mm depth, the SO_2_ value dropped significantly below the average of the healthy control group at T1, while it stayed above the average of the control group at 3 mm. This finding is in line with Koch et al. [15], where the administration of norepinephrine decreased the fracture SO_2_ value even further, while it did not affect the 3 mm measurement. The BF stayed elevated longer with a greater distance to the average of the healthy control group at 10 mm than at 3 mm. The rHb remained similarly elevated above the average of the controls at 3 and 10 mm despite the lower control average value at 10 mm than at 3 mm. However, these differences were minimal.

### 4.4. Nonunion

The reported nonunion rate for fibular fractures ranges from 0.3% to as high as 14% [41,42]. The nonunion case reported in the present study was associated with a markedly low SO_2_ value and elevated BF. In tibial fractures, SO_2_ values in nonunions have been reported to decrease on average [2,3]. However, although some patients demonstrated extremely low SO_2_ values, others exhibited increased oxygen levels, which may be due to differences in nonunion types [1]. The present nonunion case (which was observed to be associated with decreased SO_2_ levels) represents the hypotrophic type exhibiting likely decreased perfusion. Specifically, the low SO_2_ value may be a suitable parameter for detecting cases that will develop into nonunion cases at early points in time. Cases demonstrating very low SO_2_ values should at least be observed more closely and may be addressed by using one of the available noninvasive interventions, such as LIPUS, ESWT or magnetic field therapy [8,9,10]. Studies involving data from many nonunion cases are needed to investigate how SO_2_ and BF are related to nonunion types, as well as to determine the true predictive value.

### 4.5. Outliers and Confounding Factors

Although the majority of patients followed the described SO_2_ pattern, three outliers exhibited sustained high SO_2_ levels or a subsequent increase in these levels. Notably, two of these cases involved AO B3-type fractures requiring extensive surgery, specifically involving trimalleolar and syndesmotic fixation (including posterior malleolar plating). Regarding the acute phase, the absence of an initial decrease in SO_2_ in these patients may be attributable to severe soft tissue swelling associated with extensive damage that likely induced intense inflammatory hyperemia in the soft tissue envelope. The 10 mm probe may have measured parameters in the soft tissues and possibly the periosteum instead of the bone itself, where superficial hyperemia may have masked the underlying bone ischemia [43,44]. Regarding the subacute phase, micromotion associated with comminution of the fracture site may have triggered secondary healing. This process involves active callus formation and angiogenesis, which explains the sustained increase or delayed increase in SO_2_ [30,34]. Apart from swelling and the implant type, additional potential confounding factors are scar tissue formation with altered optical density and microcirculation, changes in skin thickness, differences in the approach and local tissue handling during plate fixation, and local temperature. In addition, venous and arterial diseases, as well as medications, may influence local blood supply.

### 4.6. Monitoring of Fracture Healing Progress and Speed

The first studies demonstrating distinct longitudinal SO_2_ and BF trajectories in human tibial fractures revealed the possibility that NIRS [2] or combined laser Doppler and white-light spectroscopy [3] are potentially methods for monitoring fracture healing that may support clinical decision-making. As current methods to monitor fracture healing require X-radiation, a noninvasive monitoring method that does not require ionizing radiation is highly desirable, especially for monitoring progress and healing speed [45]. However, the data obtained from fibular fractures in this study did not demonstrate a distinct trajectory comparable to the trajectories observed in tibial fractures. The prolonged SO_2_ plateau observed in this study did not return to a normal value after 6 months. Thus, in this respect, this method does not at least seem to be faster than the monitoring of fracture healing progress via radiography. Instead, its clinical value may be that it supports the earlier prediction of nonunion, at least in some cases. Additional and larger studies are needed to investigate if this is possible. Whether prediction is possible as well as which types of nonunion may be detected at early points in time need to be prospectively investigated in larger cohorts.

In the development of smart implants for fracture treatment, the new monitoring method may be of relevance to study improvements in healing speed and effects of actuation mechanisms [46]. In addition, NIRS could be used as an on-board sensing option for a smart implant [3].

### 4.7. Limitations

Several limitations of this exploratory study must be acknowledged. First, the sample size was relatively small (*n* = 14), which precluded multivariate analysis to adjust for potential confounding factors such as smoking, diabetes mellitus, and arteriosclerosis [47,48]. Thus, the specific impacts of these factors on the longitudinal SO_2_ trajectory could not be statistically isolated in this study. Larger cohorts are needed to clarify the influences of these risk factors and to confirm the findings. Second, the actual measurement depth (10 mm) of the O2C probe is unknown. In addition, the size of the measurement volume cannot be precisely reported, as it depends on tissue optical density. These factors are known limitations of this device and technology [47]. Additionally, interindividual anatomical differences (e.g., subcutaneous fat thickness or vascular status), medications and intraindividual changes over time (e.g., acute postoperative swelling) may have affected the data. Another potential limitation involves the significant difference in age between the patient and healthy control groups. Although no significant correlation between age and perfusion parameters in the healthy group was detected, the potential influence of age-related vascular changes on the healing process cannot be completely ruled out. However, the present finding is in line with the studies by Nowicki et al. [2] and Scholz et al. [3] that likewise did not find an age effect. Sex effects on bone oxygenation have not been previously reported either. However, sex differences have been reported for bone microvascular flow [49], which warrants further investigation. Effects of the body height on bone oxygenation have not been reported. However, such effects are possible based on the findings that exposure to head-down tilt and lower body negative pressure altered microvascular flow in healthy participants [50]. Myogenic vasoconstriction seems to increase bone perfusion when lowering the leg [51]. Therefore, effects of body height on bone oxygenation should similarly be studied further. Furthermore, the findings of this study are specific to closed fractures treated with ORIF and may not apply to open fractures or conservative treatment. Finally, the observation period was limited to 6 months. At this endpoint, SO_2_ remained at a low plateau, and both BF and rHb remained elevated. Due to the fact that cortical remodeling and vascular normalization may occur for years [52], future long-term studies are warranted to determine the timeline for complete physiological restitution.

## 5. Conclusions

This exploratory study was the first to assess the longitudinal perfusion trajectory of human distal fibular fractures treated with plate fixation. A decrease in SO_2_ followed by a plateau at reduced SO_2_ levels was detected. This finding markedly contrasts with the rapid increase in SO_2_ following a decrease and minimum level during recovery that has been previously reported in tibial fractures, which likely reflects anatomical differences or the distinct physiology of primary healing under absolute stability. Given that the nonunion case demonstrated markedly low SO_2_ values, due to its potential clinical relevance, this finding needs to be studied in more detail and in larger cohorts. Combined laser-Doppler and white-light spectroscopy may support the development of smart implants by serving as a tool to show differences in the healing process *in vivo*. Larger studies with more longitudinal data and nonunion cases are needed to gain more insights and evidence regarding the reported phenomena.

## Figures and Tables

**Table 1:** Characteristics of patients and the healthy control group. # Calculated by using the Mann–Whitney U test; ‡ calculated by using Fisher’s exact test.

	Patients (*n* = 14)	Healthy Control Group (*n* = 42)	*p* Value
Sex (M/F)	4/10	25/17	0.065 ^‡^
Mean age (years) ± SD	52.1 ± 15.9	39.1 ± 13.3	**0.011 ^#^**
Mean height (cm) ± SD	169.1 ± 6.9	175.5 ± 7.3	**0.006 ^#^**
Mean weight (kg) ± SD	74.9 ± 12.2	76.9 ± 12.9	0.842 **^#^**
Mean BMI (kg/m^2^) ± SD	26.1 ± 3.2	25.0 ± 4.0	0.198 **^#^**
Smoking, *n* (%)	3 (21.4)	2 (4.8)	0.094 ^‡^
Diabetes, *n* (%)	2 (14.3)	1 (2.4)	0.151 ^‡^
Time to surgery (days)	6.6 ± 2.5		
AO Classification, *n* (%)			
44-B1	6 (42.9)		
44-B2	4 (28.6)		
44-B3	4 (28.6)		
Syndesmotic fixation, *n* (%)	9 (64.3)		

**Table 2:** Characteristics and findings of the nonunion patient case.

Parameter	Value
Age at injury (years)	58
Body height (cm)	185
Body weight (kg)	98
Smoking	No
Diabetes	No
AO Classification	44-B2
SO_2_ (3 mm; 10 mm), (%)	47.66; 8.33
rHb (3 mm; 10 mm), (AU)	86; 51
BF (3 mm; 10 mm), (AU)	71.66; 157.33

**Table 3:** Average values and SD of SO_2_, BF and rHb in the healthy control group. Independent *t*-Tests were conducted if data were normally distributed (*). In case of non-normality, the Mann–Whitney U test was performed (#).

Parameter	3 mm	10 mm	*p* Value
SO_2_ (%) ± SD	39.15 ± 9.50	45.63 ± 13.68	0.012 *
rHb (AU) ± SD	69.45 ± 9.08	39.86 ± 6.53	<0.001 #
BF (AU) ± SD	23.83 ± 6.26	61.76 ± 18.90	0.263 *

## Data Availability

The datasets generated and/or analyzed during the current study are available from the corresponding author on reasonable request. Access may be granted based on a collaboration agreement. The requesting institution needs to fall within the eligibility criteria of German data protection law.

## Supplementary Materials

The following supporting information can be downloaded at: https://www.mdpi.com/article/10.3390/jfb17060306/s1, Supplementary Figure S1. Individual longitudinal trajectories of 10 mm SO_2_.

## Abbreviations

The following abbreviations are used in this manuscript:

BF	Blood flow
CT	Computed tomography
ESWT	Extracorporeal shock-wave therapy
IQRs	Interquartile ranges
LIPUS	Low-intensity pulsed ultrasound
LMM	Linear Mixed Model
NIRS	Near-infrared spectroscopy
NU	Nonunion
O2C	Name of commercial device, acronym for ‘oxygen to see’
ORIF	Open reduction and internal fixation
rHb	Relative haemoglobin
SDs	Standard deviations
SO_2_	Oxygen saturation
