# Supplementary material for: Sustained Decrease in Oxygen Saturation in Human Fibular Fractures Monitored with Laser-Doppler and White-Light Spectroscopy: A Longitudinal Observational Pilot Study
Source: J Funct Biomater. 2026 Jun 22;17(6):306. doi: 10.3390/jfb17060306 (PMC13301115; doi:10.3390/jfb17060306)
Supplement: Supplementary file 1 [file jfb-17-00306-s001.zip › jfb-4307920-supplementary.pdf]

Supplementary material

Sustained decrease in oxygen saturation in human fibular fractures monitored with laser-Doppler and white-light spectroscopy: A longitudinal observational pilot study

Tokio Kawamura, MD, Selma Fensel, MD, Marcel Orth, MD, PhD, Emmanouil Liodakis, MD, PhD, Yohei Yanasigawa, MD, PhD, Bergita Ganse, MD, PhD

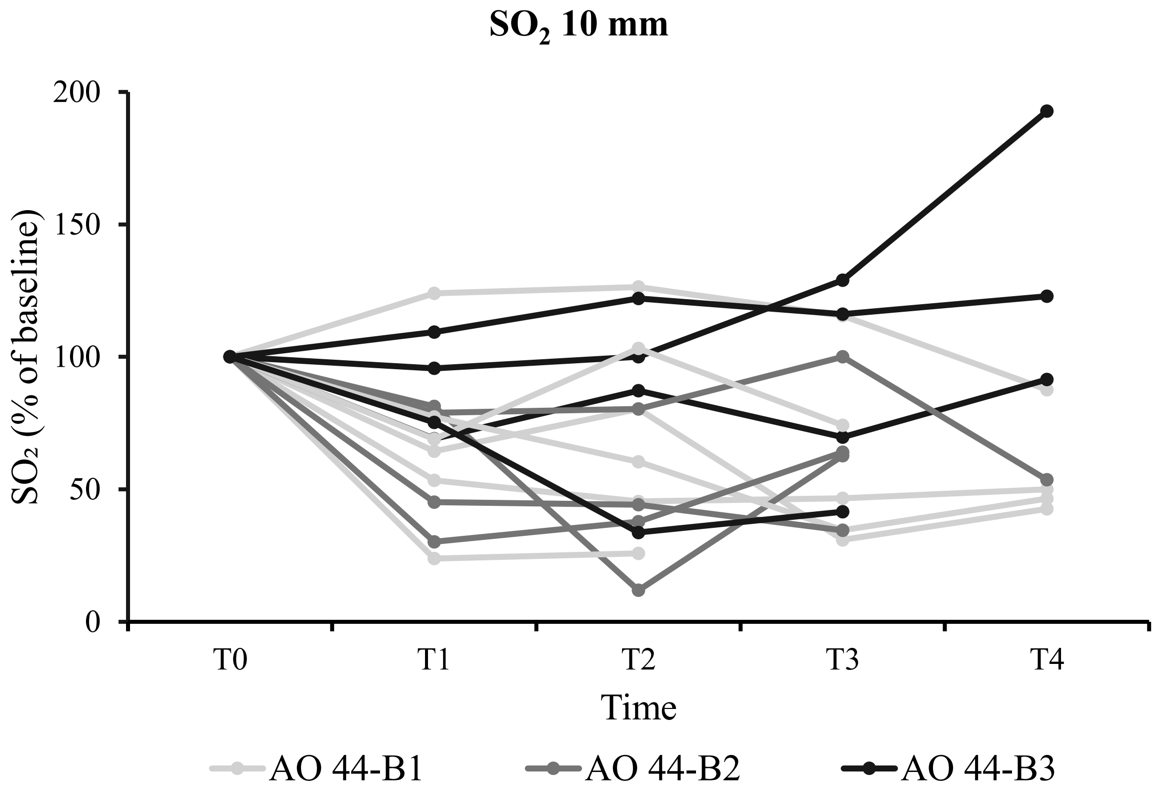

Supplementary Figure S1. Individual longitudinal trajectories of 10mm SO_2 (normalized to T0 = 100%). The trajectories are distinguished by line color to visualize the relationship between fracture severity and hemodynamic patterns. Line Color: Lines are color-coded based on AO classification: light gray (AO 44-B1), dark gray (AO 44-B2), and black (AO 44-B3). Notably, two of the three trajectories that do not decline appear in black, corresponding to the most severe fracture type (44-B3) requiring trimalleolar and syndesmotic fixation.
